# Childhood-onset RASopathy-associated hypertrophic cardiomyopathy, diastolic dysfunction, and arrhythmias

**DOI:** 10.1093/eurheartj/ehaf1012

**Published:** 2025-12-19

**Authors:** Olga Dimitra Boleti, Sotirios Roussos, Emanuele Monda, Gabrielle Norrish, Ella Field, Elena Cervi, Athanasios Bakalakos, Precylia Fernandes, Karen McLeod, Maria Ilina, Bernadette Khodaghalian, Caroline Jones, Fuensanta Escudero, Fransisco Castro, Mohamed Najih Liaqath Ali, Tara Bharucha, Gauri Nepali, Vinay Bhole, Grazia Delle Donne, Elspeth Brown, Juan Ramon Gimeno, Perry Mark Elliott, Cordula Wolf, Giuseppe Limongelli, Juan Pablo Kaski

**Affiliations:** Institute of Cardiovascular Sciences, University College London, 62 Huntley St, London WC1E 6DD, UK; Centre for Inherited Cardiovascular Diseases, Great Ormond Street Hospital, Great Ormond Street, London WC1N 3JH, UK; Department of Hygiene, Epidemiology and Medical Statistics, Medical School, National and Kapodistrian University of Athens, Athens, Greece; Inherited and Rare Cardiovascular Diseases, Department of Translational Medical Sciences, University of Campania ‘Luigi Vanvitelli’, Monaldi Hospital, Naples, Italy; European Reference Network for Rare, Low Prevalence, or Complex Disease of the Heart (ERN GUARD Heart); Institute of Cardiovascular Sciences, University College London, 62 Huntley St, London WC1E 6DD, UK; Centre for Inherited Cardiovascular Diseases, Great Ormond Street Hospital, Great Ormond Street, London WC1N 3JH, UK; Centre for Inherited Cardiovascular Diseases, Great Ormond Street Hospital, Great Ormond Street, London WC1N 3JH, UK; Centre for Inherited Cardiovascular Diseases, Great Ormond Street Hospital, Great Ormond Street, London WC1N 3JH, UK; Institute of Cardiovascular Sciences, University College London, 62 Huntley St, London WC1E 6DD, UK; Inherited Cardiac Conditions, Barts Heart Centre, London, UK; Department of Paediatric Cardiology, Royal Hospital for Children, Glasgow, UK; Department of Paediatric Cardiology, Royal Hospital for Children, Glasgow, UK; Department of Paediatric Cardiology, Royal Hospital for Children, Glasgow, UK; Department of Paediatric Cardiology, Alder Hey Hospital, Liverpool, UK; Department of Paediatric Cardiology, Alder Hey Hospital, Liverpool, UK; Pediatric Cardiology Department, Virgen de la Arrixaca Hospital, Murcia, Spain; Pediatric Cardiology Department, Virgen de la Arrixaca Hospital, Murcia, Spain; Department of Paediatric Cardiology, Southampton General Hospital, Southampton, UK; Department of Paediatric Cardiology, Southampton General Hospital, Southampton, UK; The Heart Unit, Birmingham Children’s Hospital, Birmingham, UK; The Heart Unit, Birmingham Children’s Hospital, Birmingham, UK; Department of Paediatric Cardiology, Leeds General Infirmary, Leeds, UK; Department of Paediatric Cardiology, Leeds General Infirmary, Leeds, UK; Pediatric Cardiology Department, Virgen de la Arrixaca Hospital, Murcia, Spain; Institute of Cardiovascular Sciences, University College London, 62 Huntley St, London WC1E 6DD, UK; Inherited Cardiac Conditions, Barts Heart Centre, London, UK; European Reference Network for Rare, Low Prevalence, or Complex Disease of the Heart (ERN GUARD Heart); Department of Congenital Heart Defects and Pediatric Cardiology, German Heart Center Munich, TUM University Hospital, School of Medicine & Health, Technical University of Munich, Munich, Germany; Inherited and Rare Cardiovascular Diseases, Department of Translational Medical Sciences, University of Campania ‘Luigi Vanvitelli’, Monaldi Hospital, Naples, Italy; European Reference Network for Rare, Low Prevalence, or Complex Disease of the Heart (ERN GUARD Heart); Institute of Cardiovascular Sciences, University College London, 62 Huntley St, London WC1E 6DD, UK; Centre for Inherited Cardiovascular Diseases, Great Ormond Street Hospital, Great Ormond Street, London WC1N 3JH, UK

**Keywords:** Hypertrophic cardiomyopathy, Paediatric, RASopathies, Noonan, Disease progression, Longitudinal

Key findings

- Serial data of an international cohort of 201 children with RASopathy-associated hypertrophic cardiomyopathy.
- Progressive left atrial enlargement, diastolic dysfunction, stable left ventricular hypertrophy and left ventricular outflow tract gradient, improvement in right ventricular outflow tract gradient
- A fifth of patients followed up into adulthood developed complex atrial arrhythmias in early adulthood.
- New York Heart Association/Ross functional Class >I was a time-independent predictor of major adverse cardiac events.

## Introduction

The RASopathies account for ∼18%^1^ of childhood hypertrophic cardiomyopathy (HCM) cases and up to 42%^2^ of infantile (<1 year of age) presentations, making them the second commonest cause of paediatric HCM.^1^ RASopathy-associated HCM (RAS-HCM) differs clinically^3,4^ from sarcomeric HCM, with almost 60% first-year mortality.^2,5^ Small reports have suggested spontaneous regression of left ventricular (LV) hypertrophy (LVH) in up to 17% and progression in 34%.^3^ Whether this reflects true remodelling or relative changes during growth is unclear. This study describes long-term phenotype changes in a large multicentre childhood-onset RAS-HCM cohort.

## Methods

### Study population

Retrospective data from patients <18 years with HCM^6^ and a clinical/genetic RASopathy^4,5^ diagnosis from January 1985 to December 2023 were collected using a predefined data extraction tool. Exclusions were missing baseline data or <2 follow-ups.

### Echocardiographic assessment

Echocardiographic assessment was performed as previously described.^5^ Systolic dysfunction was defined as LV ejection fraction (LVEF) < 55%, hyperdynamic function as LVEF >70% and diastolic impairment as the presence of any of: mitral valve (MV) *E*/*A* < 0.75, MV E-wave deceleration time > 240 ms, or average of lateral/septal *E*/*e*′ > 14.^7^

### Outcomes

Patients were followed every 6–12 months. Outcomes are reported at baseline, 1, 2, 5, 10 and 20 years.

The primary outcome was a composite of major adverse cardiac events (MACE): sudden cardiac death (SCD) or equivalent event, hospitalization due to congestive heart failure (CHF) symptoms, or cardiac transplantation. SCD equivalent event was defined as appropriate implantable cardioverter-defibrillator therapy, aborted cardiac arrest, or sustained ventricular tachycardia with haemodynamic compromise.

### Statistical analysis

Continuous variables are presented as median (interquartile range) or mean ± standard deviation as appropriate and categorical variables as frequencies (percentages). Between-group comparisons utilized Mann–Whitney *U* test or Student’s *t*-test for continuous variables and *χ*^2^ test or Fisher’s exact test for categorical variables.

Disease progression was assessed using mixed-effects models with random intercepts and slopes, accounting for within-subject correlation and between-centre variability.

Time-to-event analyses employed Kaplan–Meier methods and Cox proportional hazards models. Variables for multivariable models were selected based on clinical relevance and univariate *P* < .10. The final model includes all significant variables at *P* < .10.

Statistical analyses were performed using Stata version 18.0 (StataCorp, College Station, TX, USA). Two-sided *P* < .05 was considered significant, without adjustment for multiple comparisons in secondary analyses.

## Results

### Population

Of 217 patients, 201 met the inclusion criteria. Noonan syndrome was most prevalent (*n* = 155, 77.1%), and *PTPN11* was the most frequent gene (*n* = 68, 33.8%). Median age at baseline was 1.01 years (0.35–4.62). Sixty-seven patients (33.3%) had ≥1 congenital heart defects (41% valvulopathy, 10% atrial septal defect, 7% ventricular septal defect), 49 (24.6%) presented with CHF symptoms [New York Heart Association (NYHA)/Ross functional Class > I], 99 (51.3%) were taking ≥1 cardiac medications, 84 (48.6%) had biventricular involvement, and 39 (28.1%) had significant LV outflow tract (LVOT) obstruction.

### Survivors vs non-survivors

Non-survivors were younger [0.3 (0.3–1.0) years vs 1.2 (0.4–5.4) years, *P* = .019] and smaller at baseline assessment [body surface area 0.3 m^2^ (0.3–0.4) vs 0.4 m^2^ (0.3–0.7), *P* = .020]. At 1-year follow-up, a higher proportion of non-survivors was symptomatic [NYHA/Ross > I *n* = 6 (40.0%) vs *n* = 15 (13.3%), *P* = .009] and on cardiac medication [*n* = 13 (86.7%) vs *n* = 60 (53.6%), *P* = .015] and had a higher LV posterior wall thickness [7.5 mm (6.0–10.2) vs 6.1 mm (4.9–9.0), *P* = .004].

### Outcomes and predictors

Median follow-up was 7.3 years (3.1–12.6), during which 42 patients (18.3%) had MACE (incidence 1.401/100 patient-years) and 16 (7.0%) had a SCD or equivalent event (incidence 0.577/100 patient-years).

On backwards elimination multivariable analysis, NYHA/Ross > I was an independent predictor of MACE [hazard ratio: 7.08 (95% confidence interval: 1.1–43.9) *P* = .035].

### Complex atrial arrhythmias

Among 20 patients (9.9%) followed up >18 years, 4 (20%) had an episode of complex atrial arrhythmia [paroxysmal atrial fibrillation (*n* = 2), flutter (*n* = 1) or prolonged re-entrant atrial tachycardia (*n* = 1)] at a median age of 22.6 years (22.2–24.5), all had dilated atria, and 3 had moderate mitral regurgitation and elevated average *E*/*e*′ at the time.

### Phenotypic progression in survivors

**Figure 1:**
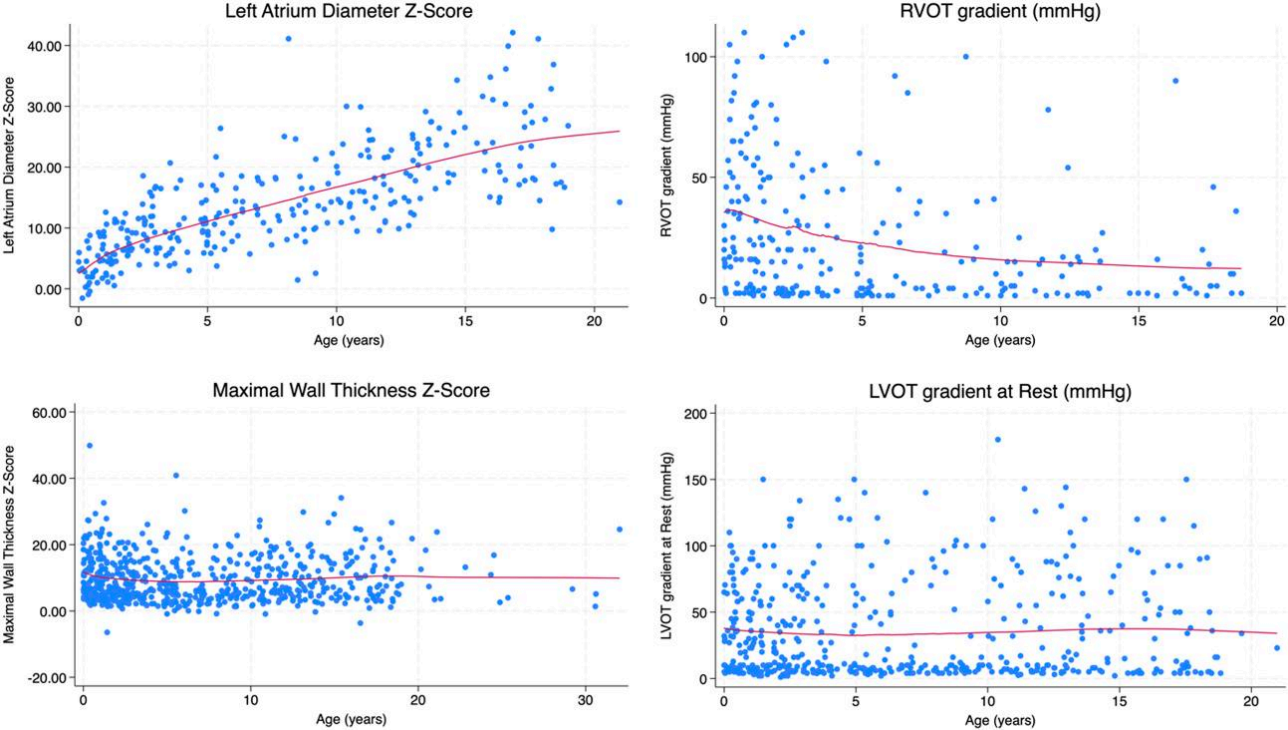
Progressive changes in left atrial diameter (*z*-score), maximal left ventricular wall thickness (*z*-score), and left and right ventricular outflow tract gradients in childhood RASopathy-associated hypertrophic cardiomyopathy with increasing age (years).

Overall, symptoms improved [NYHA > I 20.9% at baseline vs 15.4% at 10 years, *P* = .009] while medication use rose [49.4% vs 56.5%, *P* = .015]. Maximal LV wall thickness *z*-score declined [+10.3 vs +8.9 at 20 years, *P* = .039], and LVOT gradients [23 vs 7 mmHg, *P* = .019] and right ventricular outflow tract (RVOT) gradients [17 vs 5 mmHg, *P* = .001] decreased. Left atrial diameter (LAd) *z*-score worsened [+10.6 vs +25.7 at 20 years, *P* < .001] (*Figure 1*). Mixed-effects predicted annual LAd +1.17 (*P* < .001), *E*/*e*′ + .39 (*P* = .047), RVOT gradient −1.25 mmHg (*P* < .001). In a sub-analysis of the genotyped patients (*n* = 172, 78.9%), these findings were unchanged.

### Symptomatic neonates

Fifteen patients (7.5%) presented at baseline assessment with significant symptoms of CHF (NYHA/Ross functional Class III–IV) with a median age at baseline of 0.4 years (0.0–1.0), of whom five (33.3%) died. Non-survivors had a significantly smaller LV end-diastolic diameter *z*-score [−4.2 (0.1) vs −0.9 (1.0), *P* = .023] compared to surviving patients.

## Discussion

The major finding in this large, multicentre study is the demonstration of progressive LA dilatation and diastolic impairment associated with complex atrial arrhythmias in early adulthood. This novel finding suggests that similar vigilance and early consideration of anticoagulation, as in adults with sarcomeric HCM,^8^ may be appropriate and worthy of further investigation.

Another novel finding is that of CHF symptoms as a time-independent predictor of MACE in RAS-HCM. As the NYHA/Ross functional class assessment is a reproducible clinical tool, a change in functional status should prompt closer surveillance and management.

Symptomatic, non-surviving neonates had significantly smaller LV cavities. This may contribute to reduced LV stroke volume, leading to a smaller functional reserve. If validated, this finding could guide risk stratification and early treatment, including novel agents such as mTOR and MEKi.^9^ Of note, none of the patients in this study had received treatment with these agents.

### Study limitations

As a retrospective study, some data—particularly genetics—were missing.^5^ This cohort, albeit the largest of its kind, was not sufficiently powered to allow subgroup analyses for predictors of outcome and phenotypic progression. Systemic features of the disease were not analysed. Adult and neonatal subgroups were small, limiting conclusions.

## Conclusion

Patients presenting with RAS-HCM in childhood develop progressive diastolic dysfunction and LA dilatation, resulting in complex atrial arrhythmias in early adulthood. NYHA/Ross functional Class > I is an independent predictor of MACE.

## Data Availability

The data are available from the corresponding author upon reasonable request.
